# Valorization of winery and distillery by-products by hydrothermal carbonization

**DOI:** 10.1038/s41598-021-03501-7

**Published:** 2021-12-14

**Authors:** Marco Barbanera, Alessandro Cardarelli, Eleonora Carota, Marco Castellini, Tommaso Giannoni, Stefano Ubertini

**Affiliations:** 1 Department of Economics Engineering Society and Business Organization (DEIM), University of Tuscia, Largo dell’università s.n.c., Loc. Riello, 01100 Viterbo, Italy; 2 Department for Innovation in Biological, Agro-food and Forest Systems (DIBAF), University of Tuscia, 01100 Viterbo, Italy; 3 CIRIAF—Biomass Research Centre, University of Perugia, Via G. Duranti 63, 06125 Perugia, Italy

**Keywords:** Renewable energy, Energy

## Abstract

This work aims at finding an alternative strategy to manage the waste generated by the winemaking industry to obtain a solid biofuel and phenolic compounds. The effect of temperature (180–260 °C), residence time (1–7 h), and biomass-to-liquid ratio (0.05–0.25) on the co-hydrothermal carbonization of vine pruning and exhausted grape pomace, by using vinasse as moisture source, is studied. The effect of the variables is investigated and optimized using the Box–Behnken design of response surface methodology to maximize mass yield, fuel ratio, energy densification yield and phenols extraction yield and to minimize energy consumption. The statistical analysis shows that the carbonization temperature is a crucial parameter of the process, decreasing the product yield on one hand and improving the quality of hydrochar on the other. At the optimal conditions (246.3 °C, 1.6 h, 0.066), an hydrochar yield of 52.64% and a calorific value of 24.1 MJ/kg were obtained. Moreover, the analysis of the H/C and O/C ratios of hydrochars demonstrates that carbonisation significantly improves the fuel properties of solid biofuel. Liquid by-products obtained from the HTC process are found to contain high concentrations of organic matter but the BOD/COD ratios suggest their potential valorization by biological methods.

## Introduction

Winemaking industry produces a huge amount of waste, both in the form of liquid and solid materials, the management of which causes economic and environmental problems. These waste, with no economic value, are produced by viticulture, wine industry and alcohol distilleries activities and their inadequate management or disposal can cause environmental burdens^1^.

Viticulture generates vine pruning, which is the main solid by-product of the in-field interventions; typically vine pruning is left or open-burned in the agricultural fields with significant emissions of pollutants, in both gas and particulate phases^2^. Wine industry is responsible for the production of fresh grape marc biomass, mainly constituted by grape skins, seeds, pulp and stalks and obtained from grape pressing after the wine production^3^. In Europe, according to the European Council Regulation (EC) 491/2009, grape marc biomass should be sent to alcohol distilleries to recover ethanol, tartrates and to produce spirit liquors^4^. However, this process produces other waste, such as a solid fraction, the exhausted grape marc (EGM), and vinasse, which is a liquid stream whose disposal and recycling represent a serious environmental problem. Actually a large fraction of EGM is simply disposed of, and then dumped or settled in ponds constituting a relevant environmental issue due to its high organic carbon content (31–54%)^5^. Otherwise, EGM is also employed as compost or animal feed, or simply discarded as natural waste in landfills. Recent studies have demonstrated that EGM can be fruitfully employed to extract high-added polyphenols, contributing to the development of the bioeconomy in the wine industry^6^. Grape phenolic compounds are of great interest for use as antioxidants, antimicrobials, and enzyme modulators^7^. The inadequate disposal of vinasse can cause salinization, sodification, and acidification of soil, due to its low pH, high biochemical oxygen demand (BOD), high chemical oxygen demand (COD) and high solid content^8^.

Italy is one of the major wine producers with an area dedicated to vine cultivation of 657.700 hectares in 2018^9^. The wine industry produces approximately 3.0 t/ha of vine pruning, with an average moisture content of 45%, and about 2.7 t/ha of fresh grape marc, with a moisture content of 60%^10,11^. Considering also that about 10–15 L of vinasse are obtained per 1 L of ethanol produced^12^, the efficient disposal of winery waste is a serious problem from an economic and environmental point of view.

However, the main drawback to the recovery of winery and distillery waste is their high moisture content which leads to an increase of transport costs and consuion of energy needed for drying before use. In this context, the hydrothermal carbonization (HTC) process could be a valuable and profitable option to produce renewable energy from winery and distillery waste. Unlike other thermochemical conversion technologies (such as pyrolysis, gasification, and combustion), HTC allows to treat the wet feedstock without prior dewatering and drying.

HTC is a promising thermal conversion process able to convert biomass into two main streams, a coal-like material with a higher carbon content and a liquid product (process water), rich in readily biodegradable organics (e.g. organic acids, furfurals, sugars, phenols), and a small amount of gaseous products, mainly containing CO_2_^13^. The HTC is conducted in a pressure vessel by applying mild temperatures (generally between 180 and 270 °C) and high pressures (approximately 2–6 MPa) to biomass in aqueous phase with reaction time from minutes to few days^14,15^. Under these conditions, wet biomass with a low calorific value is upgraded through a series of complex reactions (hydrolysis, dehydration, decarboxylation, aromatization and condensation polymerization) into a solid product, called hydrochar, with similar characteristics to natural coal in regard to elemental composition (C, H, O and N) and lower heating value (LHV), increasing thus the energetic use of the biowaste^16^.

Only few studies on hydrothermal carbonization of waste from winemaking industry are availble in literature. Basso et al.^17^ conducted several HTC tests at different temperatures and residence times on fresh grape marc, obtaining an increase of higher heating value (HHV) of the hydrochar from 43 to 54%, at 250 °C depending on residence time. Basso et al.^18^ analyzed the behaviour of grape marc constituents (seeds, skins) during HTC at several process conditions (temperature: 180, 220 and 250 °C; reaction time: 0.5, 1, 3 and 8 h). Duman et al.^19^ analyzed the effects of HTC process on fuel characteristics and combustion behavior of hydrochar obtained from vine pruning. In all these investigations distilled water is used as reaction media in order to reach the useful moisture content of the substrate.

However, the use of pure water as the liquid source cannot be considered as an environmentally sustainable practice in light of the global water scarcity. Venna et al.^20^ and Li et al.^21^ used landfill leachate as liquid source during HTC of yard waste and food waste, highlighting that it had minimal impact on the fuel characteristics of the hydrochars than distilled water. Also, Weiner et al.^22^ demonstrated that the use of organosolv wastewater had no negative effects on the HTC process of chaff and hydrochar properties. In this way, liquid waste effluents, such as vinasse, could be an interesting option which should be explored to analyze its influence on the carbonization rate and the physical and chemical properties of the hydrochar.

This study aims at evaluating the efficiency of vinasse as moisture source for the co-hydrothermal conversion of vine pruning and EGM, recovering all the winery and distillery waste. An experimental design is defined, applying statistical response surface methodology (RSM), in order to determine the optimum values of the operating conditions for four response variables, mass yield, energy densification yield, fuel ratio and phenols extraction yield. Then an optimization study is carried out to find the best process conditions that lead to the maximization of all response variables and to the minimization of the energy consumption of the HTC process. The results obtained at the best process conditions are compared with those obtained using distilled water as reaction medium.

## Materials and methods

### Raw materials

Vine pruning used for the experiments is obtained from a local farm in Umbria, located in the Center of Italy, while EGM and vinasse are collected from the Bonollo distillery (Italy) after ethanol and tartaric acid extraction by distillation process. Vine pruning (moisture content as received: 42 wt%) and EGM (moisture content as received: 71 wt%) are dried at 105 °C for 24 h to remove residual moisture and obtain reliable and comparable results by tracking the exact amount of feedstock used in each HTC test. Subsequently, dried biomass are milled to a final particle size smaller than 500 μm to obtain uniform size distribution of the particles. Vinasse is also filtered with a 3 μm filter paper before its use in the HTC experiments, in order to properly evaluate the mass balance of the process.

Table 1 reports the chemical and physical characterization of vine pruning and EGM. We observe that the ultimate and proximate analysis shows a higher nitrogen and ash content in EGM compared to vine pruning. Since the nitrogen and ash removal after the HTC process is negligible^23^, the idea of combining EGM with woody biomass is reinforced in order to obtain a solid biofuel with better combustion properties. On the other hand, the high calorific value of EGM and its availability make this waste a promising feedstock for the HTC process. Table 1 summarises also the physicochemical characteristics of the vinasse.

**Table 1:** Physicochemical characteristics of vine pruning, EGM and vinasse (the values represent average of triplicates).

	Vine pruning	EGM	Vinasse
**Ultimate analysis (wt%, db)**			
C	44.52	46.71	
H	6.97	6.84	
N	0.84	2.33	
S	0.05	0.12	
O	44.10	36.54	
**Proximate analysis (wt%, db)**			
Volatile matter	76.07	71.03	
Ash	3.52	7.46	
Fixed carbon	20.41	21.51	
Lower heating value (MJ/kg, db)	17.59	19.20	
**Compositional analysis (wt%, db)**			
Cellulose	31.46	11.50	
Hemicellulose	12.37	7.31	
Lignin	30.29	43.14	
Extractives	6.27	11.83	
pH			5.18
COD (mg/L)			23,304
BOD (mg/L)			10,440
TOC (mg/L)			9710
Total N (mg/L)			231
Total Phenols (mg GAE/L)			781

### Hydrothermal carbonization

HTC tests are carried out in a 600 mL Teflon lined stainless steel Parr reactor (reactor series 4560, Moline, IL) under autogenic pressure. Before each trial, a blend consisted of a 50 wt% vine pruning to 50 wt% EGM is prepared. For each experimental run, the blend is directly mixed with vinasse according to the solid to liquid ratio by weight set by the experimental design (S:L) and loads into the vessel of the reactor.

Then, the system is sealed and the vessel is purged with nitrogen gas to remove air from the reactor. The loaded reactor is heated to the desired temperature with a heating rate of 3 °C/min and kept at that temperature for the required time. The stirring rate of the agitator is set to 200 rpm. The residence time is referred to the holding time of reactor after reaching the set temperature. The pressure within the reactor, only due to feedwater at the reaction temperature, is kept autogenic and is not monitored. Then, the reactor is quenched in the cold water up to room temperature and the slurry is filtered by a Büchner filtering system with a filter paper (Whatman filter paper, 8 μm). Hydrochars are washed with distilled water for several times and oven-dried at 105 °C for 24 h to remove residual moisture.

Dried hydrochars are weighted to calculate the mass yield of the HTC process and sealed in drying vessels until further use. The filtrate is bottled and maintains at + 4 °C until further analyses.

### Experimental design and process parameters

The optimum HTC conditions for hydrochar production and phenols extraction is determined through the Response Surface Methodology (RSM) which also allows to analyze the interactions between and amongst all the variables^24^. The Box–Behnken design (BBD) method with three factors and three levels is employed to optimize the independent process variables^25^.

Temperature (A), residence time (B), and S:L ratio (C) in the range of 180–260 °C, 1–7 h, and 0.05–0.25, respectively, are assumed as independent variables and coded at three levels (− 1, 0 and + 1), corresponding to low, mid and high level. The BBD experimental design includes a total of 15 tests, with 3 replicates at the central point useful to evaluate the experimental error (Table 2). Four dependent variables are selected as response variables (mass yield, energy densification yield, fuel ratio, and phenols extraction yield) which are calculated using the following equations:

1 $$MY(\%)=\left(\frac{MH}{MF}\right)\times 100,$$

2 $$EDY=\frac{{LHV}_{h}}{{LHV}_{f}},$$

3 $$FR=\frac{FC}{VM},$$

4 $$PEY \left(\frac{mgGAE}{g}\right)=\frac{\left(\left(TPC\times {V}_{l}\right)-\left({TPC}_{w}\times {V}_{w}\right)\right)}{MF},$$

where MY is the hydrochar yield (%), defined as the dry solid mass ratio of hydrochar (MH) to that of the initial feedstock (MF), EDY is the energy densification yield, obtained by the ratio between lower heating value of hydrochar (LHV_h_) and feedstock (LHV_f_), FR is the fuel ratio, determined by dividing fixed carbon content of the sample (FC) by its volatile matter content (VM), and PEY is the phenols extraction yield, obtained considering the total phenols content (TPC) in the liquid fraction (expressed as mg of gallic acid equivalents per mL), the volume of the liquid fraction (V_l_, expressed in mL), the total phenols content (TPC_w_) in the feedwater (expressed as mg of gallic acid equivalents per mL), and the volume of the feedwater (V_w_, expressed in mL).

**Table 2:** Experimental design with actual and codified levels and output variables of the process optimization experiments.

Run	Temperature (°C)	Time (h)	S:L ratio	MY (%)	EDY	FR	PEY (mg GAE/g)	EI (kJ)
1	260 (+ 1)	1 (− 1)	0.15 (0)	51.11	1.27	0.68	11.52	304.53
2	220 (0)	1 (− 1)	0.05 (− 1)	59.33	1.08	0.43	22.03	261.25
3	220 (0)	4 (0)	0.15 (0)	60.22	1.15	0.59	7.40	296.45
4	220 (0)	4 (0)	0.15 (0)	62.13	1.15	0.56	7.61	296.45
5	180 (− 1)	4 (0)	0.25 (+ 1)	75.73	0.99	0.40	2.22	223.65
6	220 (0)	7 (+ 1)	0.05 (− 1)	50.34	1.22	0.67	21.89	356.82
7	220 (0)	4 (0)	0.15 (0)	59.98	1.16	0.58	9.35	296.45
8	260 (+ 1)	4 (0)	0.25 (+ 1)	50.53	1.29	0.78	11.80	346.44
9	180 (− 1)	7 (+ 1)	0.15 (0)	70.44	1.09	0.43	5.90	271.43
10	260 (+ 1)	7 (+ 1)	0.15 (0)	46.67	1.35	0.80	12.92	419.70
11	260 (+ 1)	4 (0)	0.05 (− 1)	45.33	1.33	0.76	27.90	377.79
12	220 (0)	7 (+ 1)	0.25 (+ 1)	61.60	1.18	0.61	6.32	331.65
13	220 (0)	1 (− 1)	0.25 (+ 1)	65.23	1.08	0.48	8.34	236.09
14	180 (− 1)	4 (0)	0.05 (− 1)	66.05	1.03	0.39	15.80	243.24
15	180 (− 1)	1 (− 1)	0.15 (0)	70.13	0.98	0.38	3.94	195.47

*MY* hydrochar yield, *EDY* energy densification yield, *FR* fuel ratio, *PEY* phenols extraction yield, *EI* energy input.

A second order polynomial equation (Eq. (5)) is used to establish a mathematical relationship between the independent variables and the responses:

5 $$Y={\beta }_{0}+\sum_{i=1}^{3}{\beta }_{i}{X}_{i}+\sum_{i=1}^{3}{\beta }_{ii}{X}_{i}^{2}+\sum_{i=1}^{3}\sum_{j=i+1}^{3}{\beta }_{ij}{X}_{i}{X}_{j},$$

where Y is the predicted response and β_0_, β_i_, β_ii_, and β_ij_ are regression coefficients for intercept, linear, quadratic and interaction terms, respectively.

The analysis of variance (ANOVA) for the determination of significance of models and process parameters are conducted using the Minitab 17 statistical software^26^. Model validation and numerical optimization by desirability function are also performed. The goal for response in the desirability function approach is at the the same time reaching a maximum for all the selected response variables. Each variable is transformed to a dimensionless desirability value, *d*, in the range 0 ≤ *d* ≤ 1 and if *d* is equal to 0 than the response is completely unacceptable while if *d* is equal to 1 the response is at its goal^27^.

### Energy consumption of the process

A simplified calculation of the energy input for the thermal treatments is performed considering the energy required to heat water and biomass in a closed batch system and the heat loss from the reactor. The initial temperature in the reactor system is assumed to be 25 °C and heat released and absorbed by chemical reactions is neglected because HTC reactions are net exothermic based on external energy input to work at ideal temperatures^28^. The energy input (EI) at different operating conditions is calculated as follows:

6 $$EI\left( {{\text{kJ}}} \right) = \left[ {m_{{water}} \times \left( {H_{{water,HTC}} - H_{{water,25}} } \right)} \right] + \left[ {m_{{feedstock}} \times c_{{p,feedstock}} \times \left( {T_{{HTC}} - 25} \right)} \right] + \left[ {A_{s} \times {\raise0.7ex\hbox{$k$} \!\mathord{\left/ {\vphantom {k s}}\right.\kern-\nulldelimiterspace} \!\lower0.7ex\hbox{$s$}} \times \left( {T_{{HTC}} - 25} \right) \times t} \right],$$

being m_water_ (kg) and m_feedstock_ (kg) the water and biomass weight of the mixture, respectively. In Eq. (6) H_water,HTC_ and H_water,25_ are the enthalpy of water at the HTC temperature and at 25 °C, respectively, A_s_ is the surface area of the reactor (0.041 m^2^), k and s are the thermal conductivity of the insulation material (0.042 W/mK) and the thickness of the insulation (0.075 m), respectively. The specific heat capacity of the biomass, c_p,feedstock_ (J/kg K), is, calculated through Eq. (7) by assuming a mean temperature between the HTC temperature and 25 °C:

7 $${c}_{p,feedstock}=2300-1150\times {e}^{-0.0055T}.$$

Such a calculation does not account for water vaporization enthalpy because phase change is avoided in the HTC process and for drying the produced hydrochar.

### Analytical methods

#### Analysis of solid samples

The untreated and carbonized biomass are analyzed through chemical and physical characterization. Proximate analysis is performed to determine the moisture content, ash content and volatile matter according to the UNI EN ISO 18134-2:2017, the UNI EN ISO 18122:2016, and the UNI EN ISO 18123:2016 methods, respectively, through thermogravimetric analyzer (TGA-701, LECO Corporation). Fixed carbon (FC) content is obtained by subtracting the sum of moisture, ash and volatile matter from 100 wt%. Elemental analyser (TruSpec CHN, LECO Corporation) is used to analyze the carbon, hydrogen, and nitrogen contents of the samples, according to the UNI EN ISO 16948:2015. Total content of sulphur is determined following the UNI EN 15289 (method A) by using the inductively coupled plasma emission spectrometer (Optima2000DV, Perkin Elmer). The oxygen content is calculated by difference as O = 100 − (C + N + H + S + ash). HHV of samples is measured according to the UNI EN ISO 18125:2018 by means of a isoperibolic calorimeter (AC-350, LECO Corporation). Then, the lower heating value (LHV) was calculated from the higher heating value, depending on the hydrogen content. Compositional analysis, in terms of hemicellulose, cellulose, lignin, and extractives contents is performed according to the National Renewable Energy Laboratory (NREL) protocols^29^, as described in^30^.

#### Analysis of liquid samples

Feedwater and aqueous phase are characterized in terms of pH, total organic carbon (TOC), total nitrogen (TN), chemical and biological oxygen demand (COD, BOD), and TPC. pH is determined using a pH-meter (HI 2221-02, Hanna instruments). COD, BOD and TOC are determined according to Standard Methods^31^. Total nitrogen is determined by a modified Kjeldahl method ^32^ through microwave digestion (CEM Mars Xpress, Matthews) with a mixture of 37% HCl (Carlo Erba Reagenti, Italy) and 30% H_2_O_2_ (Merck KGaA, Germany) and the following spectrophotometric determination of ammonium using the nitroprusside method^30^. Total phenols content is determined by means of a spectrophotometer at 750 nm wavelenght with Folin–Ciocalteu reagent^33^. The results are expressed as mg of gallic acid per L of liquid fraction.

## Results and discussion

### Effects of process parameters

#### Effects of process parameters on mass yield

As shown in Table 2, HTC operating conditions have a significant impact on the mass yield, dispersing widely from 45.33 (260–4–0.05) to 75.73% (180–4–0.25), which suggests that the hydrochar yield depended on the choice of hydrothermal conditions.

**Figure 1:**
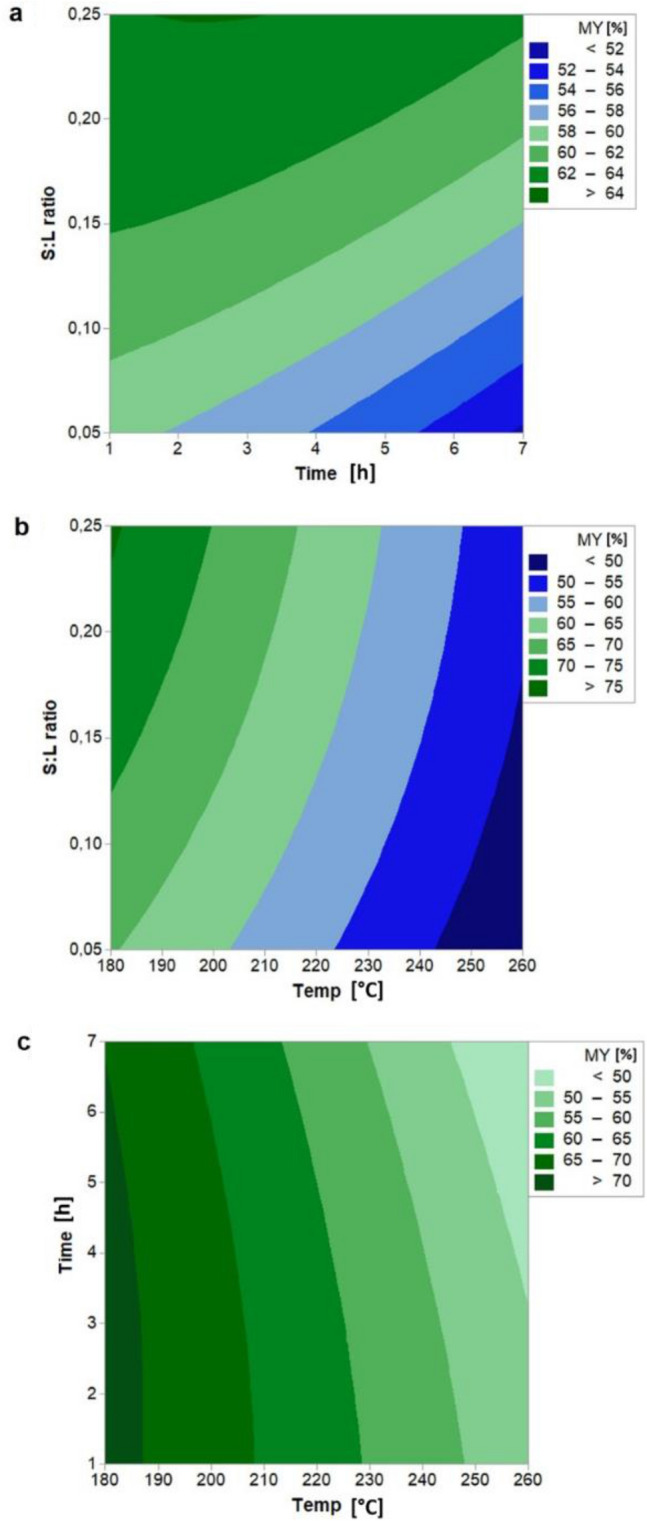
Contour plot of MY (Hydrochar Yield) with interaction between (**a**) S:L ratio and time, (**b**) S:L ratio and temperature, (**c**) time and temperature. Hold values: Temperature: 220 °C, time: 4 h, and S:L ratio: 0.15.

Figure 1 shows the contour plots of MY and highlights the interaction effects of the independent variables. We note that temperature has a high influence on hydrochar yield. In fact, as a decrease in the hydrochar yield from 70.59 to 48.41% is observed with the HTC temperature increases from 180 to 260 °C, keeping constant the other variables. This is in agreement with other studies available in literature^34,35^, which show that a temperature rise increases the decomposition of biomass, mostly due to the degradation of cellulose and hemicellulose, and the potential for the release of volatile matters. Furthermore, the decomposition of biomass could be facilitated at higher temperatures by decreasing the dielectric constant of water (i.e. by increasing the reaction temperature), making the water behaving similarly to a polar organic solvent^36^.

A negative correlation with the hydrochar yield is observed also for the residence time, although the effect is milder than that produce by the HTC temperature. This is probably ascribable to the slow heating rate which ensures sufficient time for biomass decomposition and for the cross-reaction of intermediate products, as also observed by Chen et al.^35^. Moreover, the further mass loss during the residence time, which is to say after heating up to the desired reaction temperature, may be related to the formation of lighter organic compounds and permanents gases. An opposite trend is observed for the S:L ratio, whose growth from 0.05 to 0.25 increases the mass yield from 55.26% to 63.27%, thanks to the grater ability of the liquid phase to dissolve and maintain solubilised chemical species deriving from biomass^37^.

Based on the BBD results showed in Table 2, an empirical relationship that relates the response (mass yield) and selected variables is obtained, as described by Eq. (8).

8 $$MY\left(\%\right)=86.2-0.071A+1.470B+1.112C-0.0003{A}^{2}-0.082{B}^{2}-0.009{C}^{2}-0.010AB-0.003AC+0.045BC.$$

The significance and suitability of the quadratic model is evaluated by ANOVA at 95% confidence (p < 0.05), and the results are shown in Table S1 (Supplementary Materials). The p-values and F-values are applied to evaluate the significance of the coefficients. In particular, the terms with a p-value lower than 0.050 denote a higher significance of coefficient. It is interesting to note that only the linear terms are found to be statistically significant (p > 0.05) meaning that the hydrochar yield depends more upon the individual change of the independent variables, rather than their interactions.

According to these results, the model F-value of 47.61 indicates that the model is statistically significant. The adequacy of the model is further confirmed by an adjusted determination coefficient (R^2^) of 0.9677 and a lack of fit (LOF) test 2.62. The R^2^ obtained for the regression model is 0.9885, meaning that the model explains 98.85% of the variation around the mean. However, R^2^ is sensitive to the degree of freedom and, thus, adjusted R^2^ is more reliable to be used for model evaluation, decreasing the number of insignificant terms in the model when the model factors increases. Its value higher than 0.95 proves the adequacy of the model. Furthermore, the good agreement between predicted R^2^ value of 0.8477 and adjusted R^2^ value of 0.9677 (being within 0.2 of each other) suggests that there is a linear relationship between the experimental and calculated mass yield^38^. In addition, the lack of fit test is carried out to determine the adequacy of the regression model in describing the observed data or if significant terms are excluded from the model. The lack-of-fit should be not significant (p-value > 0.05) for a suitable fitting of response surface models. The “Lack of Fit F-value” of 2.62 implies there is a 28.9% chance that a “Lack of Fit F-value” this large could occur due to noise.

#### Effects of process parameters on energy densification yield

The observed EDY values higher than 1 clearly indicate that the HTC treatment improves the energy density of the hydrochar. The increased calorific value of hydrochar EDY values is observed at all tested conditions, except for the hydrochar obtained at 180 °C—4 h—0.25 and at 180 °C—1 h—0.05. Anyway, in these cases, the EDY reduction is always below 2%, thus confirming that a good energy densification is obtained at most operating conditions.

We also note that EDY is strongly affected by the processing variables, with experimental values ranging from EDY = 0.98, at 180 °C—1 h—0.05, to EDY = 1.35, at 260 °C—7 h—0.15. Similar energy densification ratios are obtained for hydrochars produced from paper mill sludge residue (EDY = 1–1.6^39^) and spent coffee grounds (EDY = 1.16 to 1.45^40^). In contrast to the hydrochar yield trend, the energy densification yield of hydrochar grows by increasing the HTC temperature and the residence time.

**Figure 2:**
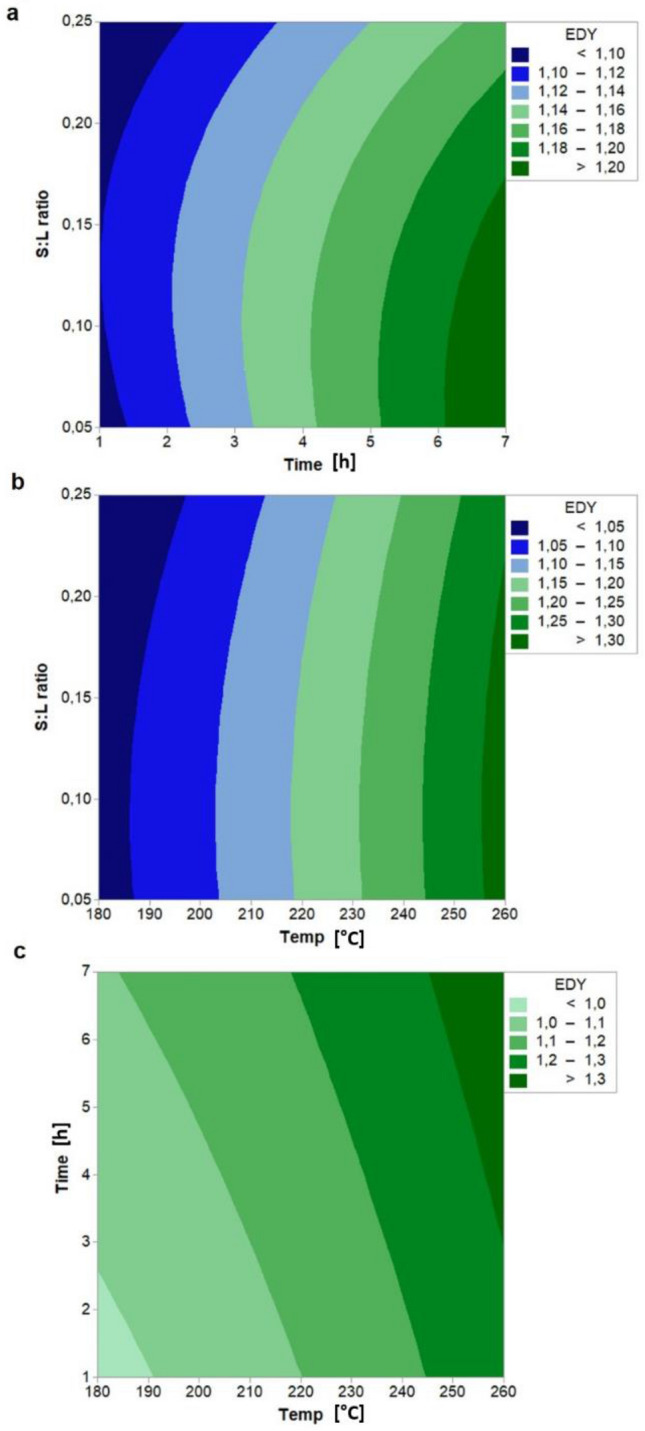
Contour plot of EDY (Energy Densification Yield) with interaction between (**a**) S:L ratio and time, (**b**) S:L ratio and temperature, (**c**) time and temperature. Hold values: Temperature: 220 °C, time: 4 h, and S:L ratio: 0.15.

The influence of the S:L ratio on EDY, shown in Fig. 2, is less significant with a slight EDY decrease detected at high and low S:L ratios for an HTC temperature below 210 °C and only at high S:L ratios in the rest of the temperature observation range.

Similar effects of temperature and residence time upon energy densification yield have been observed in literature with sunflower stalk^41^, miscanthus^42^, and almond-tree pruning^43^. In fact, under severe conditions, the increase of EDY is mainly due to the decarboxylation of biomass components that causes a reduction of the oxygen content in the hydrochar^39^.

The following equation represents the quadratic response surface regression model generated by ANOVA:

9 $$EDY=0.873-0.002A+0.042B+0.004C+0.00001{A}^{2}-0.0004{B}^{2}-0.0001{C}^{2}-0.00007AB-0.000001AC-0.0004BC.$$

Equation (9) allows to predict the EDY in terms of the actual factors. The F and p values, summarised in Table S1 (Supplementary Materials), demonstrate that the model is statistically significant as well as the linear coefficients (A, B, C) and a quadratic term coefficient (A^2^), and indicate a nonlinear relationship between the hydrochar yield and the reaction temperature.

The high value of regression coefficient (0.9959) suggests that the model could be accepted with satisfactory accuracy. The value of adjusted R^2^ (0.9885) confirms that the model is highly significant. In addition, high predicted R^2^ (0.9365) indicates that the model could be used to predict response for a given set of independent variables. The “Lack of Fit F-value” of 16.72 implies the Lack of Fit is not significant relative to the pure error. There is a 5.70% chance that a “Lack of Fit F-value” this large could occur due to noise.

#### Effects of process parameters on fuel ratio

The fuel ratio is used to understand the combustibility of the solid fuel; high values of the fuel ratio indicate a more stable combustion and a less violent flame, while low values suggest incomplete combustion with a higher smoke emission tendency^44^.

**Figure 3:**
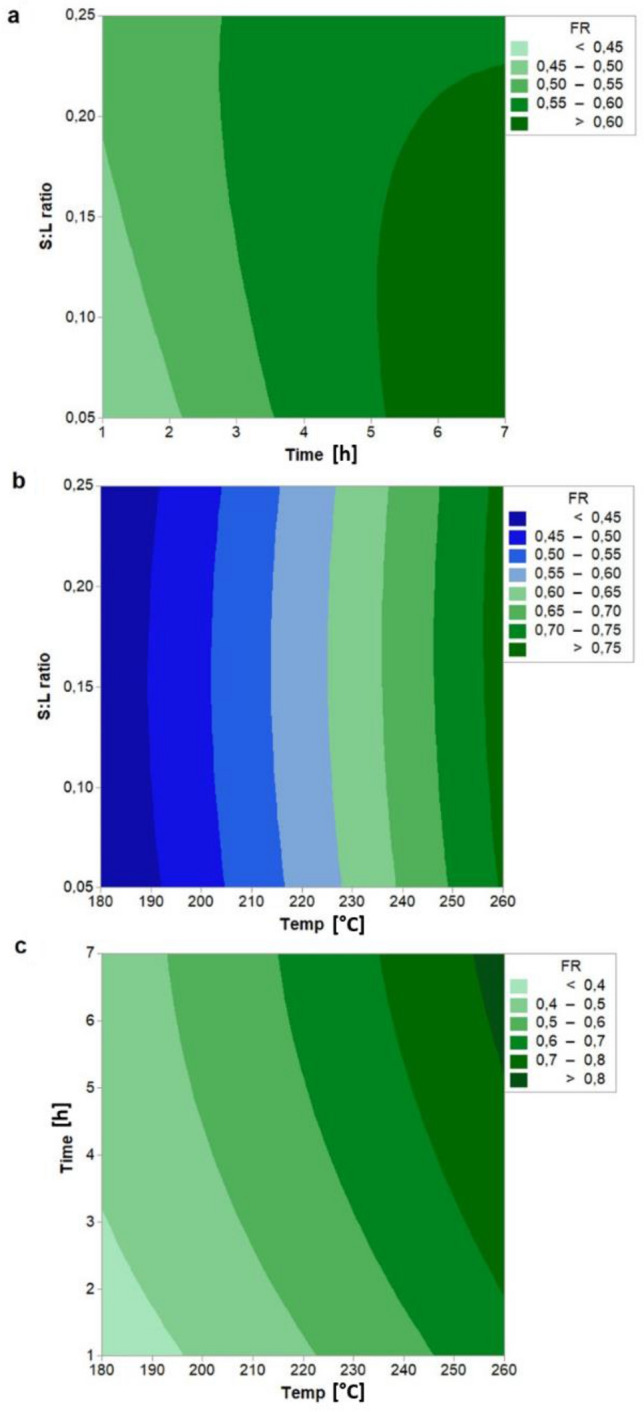
Contour plot of FR (Fuel Ratio) with interaction between (**a**) S:L ratio and time, (**b**) S:L ratio and temperature, (**c**) time and temperature. Hold values: Temperature: 220 °C, time: 4 h, and S:L ratio: 0.15.

Figure 3 shows the effect of temperature, residence time and S:L ratio on the fuel ratio. We note that the fuel ratio is influenced by the HTC temperature and the residence time while the effect of the S:L ratio is negligible. Fuel ratio is found to rise steeply with the temperature increasing from 0.40 to 0.76 (keeping constant the other variables), obtaining a maximum increase of about 121% when compared with raw feedstock. This result is mainly due to the rapid decrease of volatile matter content at a higher temperature, which accelerates the deoxygenation reaction of the biowaste. Similar trend and values have been observed for other biomass in previous studies^35,40,45^ while slightly different values have been obtained for the HTC process of the single winery and distillery by-products using distilled water as process water. Petrovic et al.^46^ obtained a fuel ratio of 0.41 from hydrothermal carbonization of grape pomace at 220 °C for 1 h, while Duman et al.^19^ reported that FR varied from 0.68 (at 220 °C for 120 min) to 1.91 (at 260 °C for 30 min) for vine pruning. The effect of the holding time on the fuel ratio is less significant than the effect of temperature.

However, it can be observed that the fuel ratio significantly increases (from 0.49 to 0.58) when the residence time rises from 1 to 4 h, while, for a residence time up to 7 h, the fuel ratio slightly increases only up to 0.63.

Regression model for fuel ratio derived from ANOVA analysis is given below:

10 $$FR=-0.013-0.0003A+0.028B+0.004C+0.000009{A}^{2}-0.002{B}^{2}-0.00009{C}^{2}+0.0001AB+0.00001AC-0.0009BC.$$

ANOVA results, presented in Table S1 (Supplementary materials), show that only the linear terms of reaction temperature and residence time of the regression model are significant. There is only a 0.1% chance that Model F-Value could occur due to noise and confirms the validity of the predicted model. The lack of fit F-value of 8.86 implies the lack of fit is not significant, as there is 10.3% chance that the lack of fit F-value occurs due to noise. The closeness of R^2^ (0.9804) and adj-R^2^ (0.9452) values shows also that there is a good relationship between the experimental and predicted value.

#### Effects of process parameters on phenols extraction yield

**Figure 4:**
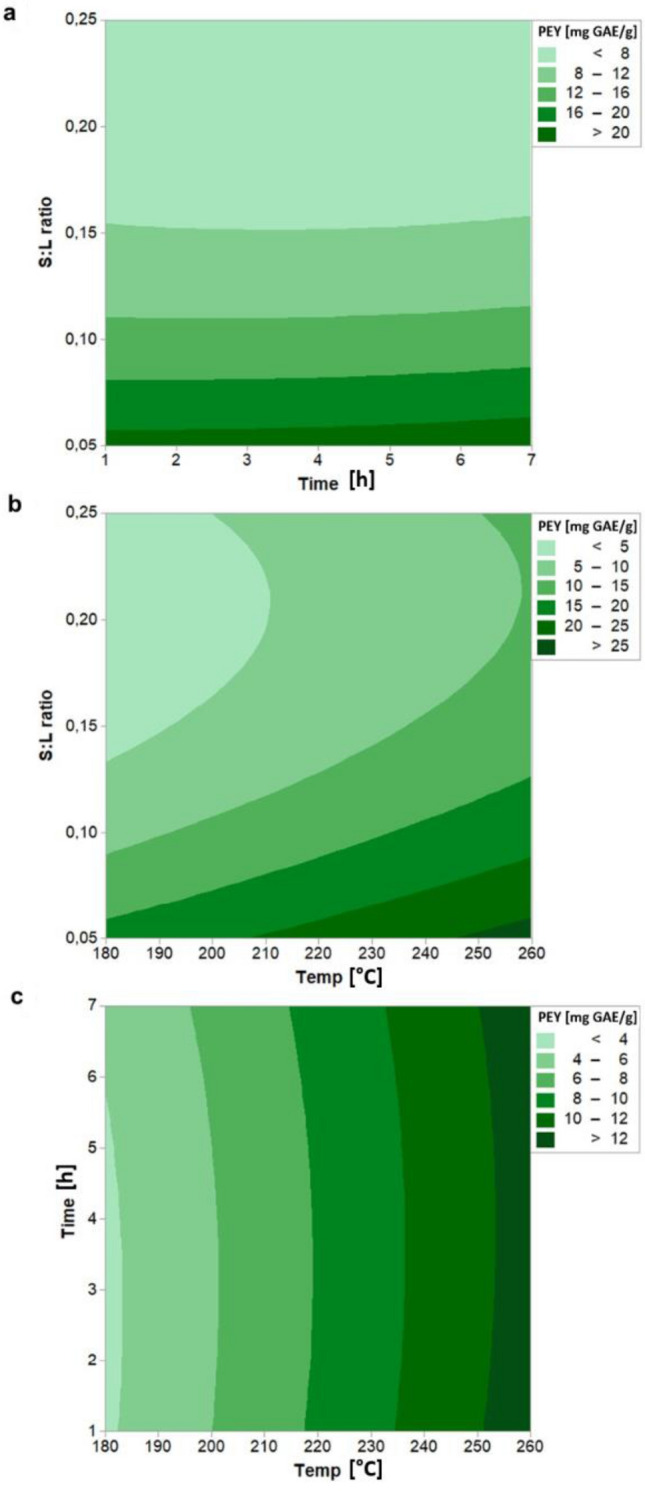
Contour plot of PEY (Phenols Extraction Yield) with interaction between (**a**) S:L ratio and time, (**b**) S:L ratio and temperature, (**c**) time and temperature. Hold values: Temperature: 220 °C, time: 4 h, and S:L ratio: 0.15.

Vine pruning and EGM are composed by various non-volatile and volatile phenolic compounds that could be interesting to extract to be used in pharmaceutical, cosmetics, and food industries. In particular, the high amount of phenolic compounds in the grape pomace is due to their partial extraction during the winemaking process; however, the TPC content is significantly reduced in the exhausted grape marc due to the thermal degradation of polyphenols during the distillation process at high temperatures^6^. Figure 4 shows that PEY is mostly influenced by S:L ratio and temperature while reaction time plays a negligible role in the polyphenols extraction. Atallah et al.^47^ obtained the same result during the hydrothermal carbonization of olive mill wastewater. Biomass-water ratio has the main influence on the PEY, resulting in a steep reduction of the PEY, with the increase of ratio from 0.05 (21.91 mg GAE/g) to 0.15 (8.38 mg GAE/g), followed by a more gradual decrease (7.17 mg GAE/g, at S:L ratio: 0.25). On the other hand, HTC temperature has a more linear effect on the EPY, causing an increase of the phenols extraction from 6.96 mg GAE/g to 16.04 mg GAE/g, when temperature raises from 180 to 260 °C. This trend could be explained by considering that phenolic compounds in biomass are both present in a soluble form (free or conjugated to soluble carbohydrates by ester/ether bonds) or in an insoluble form bound by ester/ether bonds to lignin^48^. Thus, an high water content in the HTC process allows to ease the solubilization of the free polyphenols and, under high temperature, it plays the role of reactant hydrolyzing lignocellulosic structure. In fact, lignin starts to decompose at 200 °C due to the dissociation of β-O-4 linkages by hydrolysis to produce phenol, aldehyde, and ketones, which are polar compounds diffusing into the liquid phase^49^. To obtain the maximum phenols extraction yield (27.78 mg GAE/g), time and temperature should be kept at the maximum level while solid-to-liquid ratio should be at the minimum value. This result is interesting if compared with the most common methods for recovery of polyphenols from grape pomace. Drevelegka and Goula^50^ obtained a maximum yield of 33.88 mg GAE/g by direct ultrasound-assisted extraction, Da Porto and Natolino^51^ found an optimum value of 24.14 mg GAE/g dry pomace by employing the solid–liquid extraction technique, while according to Aliakbarian et al.^52^ a yield of 31.69 mg GAE/g dry pomace was obtained by subcritical water extraction.

The regression equation of the PEY response in terms of the actual variables is as follows:

11 $$PEY\left( {{\raise0.7ex\hbox{${{\text{mg}}GAE}$} \!\mathord{\left/ {\vphantom {{{\text{mg}}GAE} {\text{g}}}}\right.\kern-\nulldelimiterspace} \!\lower0.7ex\hbox{${\text{g}}$}}} \right) = 4.900 + 0.109A + 0.240B - 2.185C + 0.00007A^{2} + 0.037B^{2} + 0.062C^{2} - 0.001AB - 0.002AC - 0.016BC.$$

The analysis of variance confirms S:L ratio as having the most significant enhancement effect on phenols extraction given by its very large *F* value (436.116). The analysis reveals that the linear terms A, C are significant model terms, as well as the squared term C^2^. The statistical analysis of the response reveals that the regression coefficient (R^2^) was 0.9836, whereas the predicted R^2^ is 0.8898 and the adjusted R^2^ is 0.9541.

The probability of F function for the model term is less than 0.05 which confirms the significance of the model, while the probability of F function for the lack of fit is greater than 0.05 which proves the non-significance of error. Furthermore, the lack of fit F-value of 2.91 shows that the lack of fit is not significant relative to pure error.

### Hydrochar properties: proximate and elemental composition

Table 3 shows the elemental composition of the hydrochar obtained under different HTC conditions. It can be noted that the carbon content significantly increases with increasing carbonisation temperature while residence time and solid-to-liquid ratio do not have a prominent effect on the C content. In addition, the HTC conditions has not statistically significant effect on the hydrogen, nitrogen and sulphur content of the hydrochar. Similar results are obtained by Tag et al.^41^ during the hydrothermal carbonization of poultry litter, sunflower stalks, and algal biomass.

**Table 3:** Proximate analysis, ultimate analysis and heating value of hydrochars.

Sample	Elemental analysis (wt%, db)							Proximate analysis (wt%, db)			LHV (MJ/kg, db)
	C	H	N	S	O	H/C	O/C	VM	Ash	FC	
180–1–0.15	49.25	6.53	1.47	0.15	39.36	1.59	0.60	70.41	3.25	26.45	18.03
180–4–0.05	51.97	7.60	2.18	0.09	34.96	1.75	0.50	69.52	3.20	27.29	18.95
180–4–0.25	50.24	7.02	2.07	0.23	36.21	1.68	0.54	68.94	3.24	27.63	18.21
180–7–0.15	53.50	7.27	2.13	0.21	33.54	1.63	0.47	67.37	3.36	29.28	20.05
220–1–0.05	55.11	7.58	2.13	0.11	31.32	1.65	0.43	67.18	3.75	29.08	19.88
220–1–0.25	55.03	6.46	1.53	0.04	33.43	1.41	0.46	65.08	3.51	31.42	19.87
220–4–0.15	60.30	6.32	1.81	0.19	26.99	1.26	0.34	61.11	4.39	34.50	21.16
220–4–0.15	59.12	6.44	1.87	0.14	28.91	1.31	0.37	60.55	3.53	35.93	21.17
220–4–0.15	59.55	6.38	1.83	0.20	28.65	1.29	0.36	61.30	3.40	35.31	21.34
220–7–0.05	60.96	7.53	2.75	0.21	23.61	1.48	0.29	56.81	4.95	38.25	22.44
220–7–0.25	60.53	7.29	2.45	0.12	25.82	1.45	0.32	59.92	3.79	36.30	21.71
260–1–0.15	66.61	6.60	2.15	0.31	20.56	1.19	0.23	57.25	3.77	38.98	23.36
260–4–0.05	65.65	7.75	3.06	0.22	16.88	1.42	0.19	53.04	5.44	41.52	24.47
260–4–0.25	69.01	6.82	2.28	0.12	18.11	1.19	0.20	53.99	3.66	42.36	23.73
260–7–0.15	69.18	6.66	2.32	0.19	17.30	1.15	0.19	53.04	4.36	42.39	24.84

*VM* volatile matter, *FC* fixed carbon, *LHV* lower heating value.

The increase of C content is mainly due to the degradation of organic constituents, hemicellulose and cellulose, and the deposition of dissolved compounds^53^. Furthermore, with the increase of the HTC temperature the dehydration and decarboxylation reactions of the coalification process are more intense, resulting in the decrease of H/C and O/C atomic ratios and, thus, in the increase of LHV and energy density of the hydrochar. In particular, the decomposition and cracking of biomass lead to the reduction of oxygen content in the hydrochar which is reduced by a maximum of 61.8% (for the sample 260–4–0.05) from the original feedstock (40.32 wt%, db, calculated as the mean value of O content of vine pruning and EGM).

**Figure 5:**
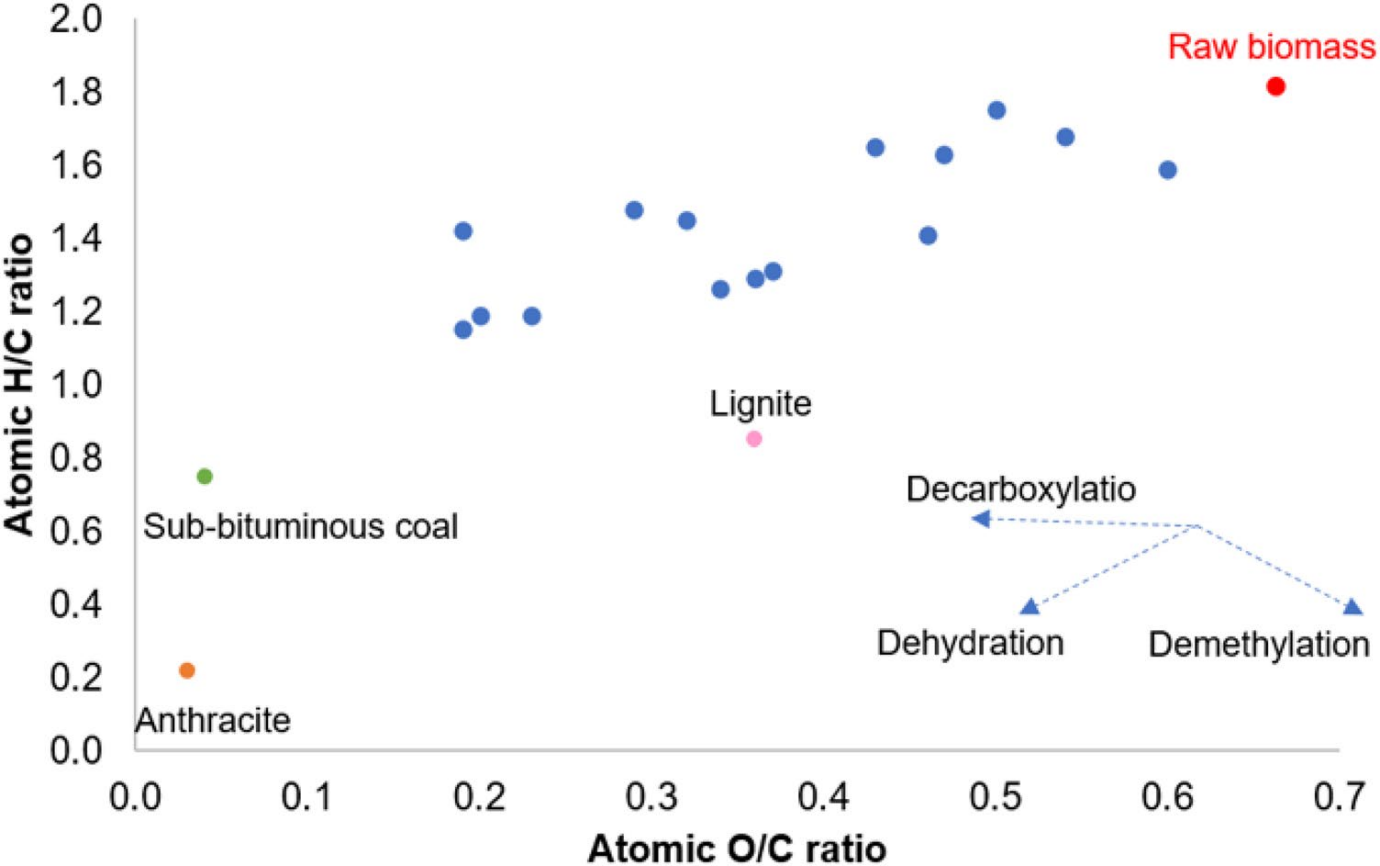
Van Krevelen diagram for hydrochars under different conditions**.**

The Van Krevelen diagram (Fig. 5) is used to analyzed the coalification degree of the produced hydrochar during the HTC process. In this plot, the dashed lines represent the dehydration, decarboxylation, and demethanation processes and other H/C–O/C atomic ratios^54^ are reported for other feedstock (anthracite, lignite, sub-bituminous coal) in order to perform a comparison with the hydrochar samples. It is clear that the predominant reaction pathways are dehydration (production of water) and decarboxylation (formation of carbonyls including carboxylic acids) while demathanation (production of methane) has a negligible influence on the HTC process. When the coalification degree of the hydrochar is compared with other typical fossil fuels, it is observed that at higher temperatures the hydrochar shows greater carbonaceous similarity with coal and lignite structure.

In particular, lower atomic ratios of the hydrochar than the raw material demonstrates the enhancement of the fuel properties of hydrochars because of the less smoke and water vapor release. The important upgrading of the fuel quality of the hydrochars is also confirmed by the LHV values which increases with increasing HTC temperature. In particular, the maximum values 23.36–24.84 MJ/kg obtained at 260 °C are comparable to that of bituminous coal, demonstrating the effectiveness of the HTC treatment of the winery and distillery by-products to produce coal substitutes.

Table 3 shows also the proximate analysis of hydrochars, from which it can be observed that reaction temperature has the main effect on the volatile matter and fixed carbon contents. The increase of HTC temperature causes a reduction of the volatile matter and an accumulation of fixed carbon, confirming that dehydration, decarboxylation as well as carbonization reactions occur during HTC treatment.

In particular, the volatiles decreases significantly starting from the temperature of 220 °C, mainly due to the fact that cellulose starts to decompose at this temperature^35^. It should be also pointed out that hydrothermal carbonization at 260 °C with residence time above 4 h allows to obtain hydrochars having volatiles in the range 53.09–53.99%, which are similar to that of coal. When the HTC temperature and residence time increase, the cross-linking reaction happens, obtaining hydrochars with higher carbon content by the elimination of H_2_O and CO_2_. As regards the ash content, the HTC treatment allows its decrease from 5.49 wt% (calculated as the mean value of ash content of vine pruning and EGM) in the raw material to 3.20–3.36 wt% at 180 °C, mainly due to the dissolution of organic and inorganic elements (especially Na+ and K+) into subcritical water, favoured by acid solvation via the acetic acid produced during the HTC process^55^. However, with the increase of the severity of the process, the ash content slightly increases maybe due to the re-precipitation of some inorganics components onto the hydrochar^16^.

### Aqueous phase composition

HTC temperature, residence time, and S:L ratio has also an essential role in the physical–chemical properties of the liquid fraction product as shown in Table 4. To understand the effects of the operating parameters on the spent liquor, the main effects plots are shown in Figs. S1–S5 (Supplementary Materials), indicating the relative strength of effects of various factors. A main effect is present when the mean response changes across the level of a factor.

**Table 4:** Characterization of process waters at different HTC conditions.

Temperature (°C)	Time (h)	S:L ratio	pH	Total N (mg/L)	TOC (g/L)	COD (g/L)	BOD (g/L)	COD/TOC	BOD/COD
180	1	0.15	3.80	352.27	12.71	27.72	14.91	2.18	0.54
180	4	0.05	4.10	339.39	12.63	29.28	11.96	2.32	0.41
180	4	0.25	3.47	549.53	20.82	47.98	21.61	2.30	0.45
180	7	0.15	3.83	455.90	17.51	38.57	19.42	2.20	0.50
220	1	0.05	4.08	368.36	12.06	28.43	8.87	2.36	0.31
220	1	0.25	3.83	603.54	21.66	52.75	22.33	2.44	0.42
220	4	0.15	4.13	331.42	13.89	27.38	6.31	1.97	0.23
220	4	0.15	4.03	356.58	14.01	30.18	7.55	2.15	0.25
220	4	0.15	4.33	350.07	13.56	29.54	7.34	2.18	0.25
220	7	0.05	4.09	394.36	16.91	28.00	14.77	1.66	0.53
220	7	0.25	4.28	366.35	23.69	56.02	11.99	2.36	0.21
260	1	0.15	4.52	452.65	12.72	24.99	15.41	1.96	0.62
260	4	0.05	4.40	323.73	13.42	32.97	13.92	2.46	0.42
260	4	0.25	3.96	681.47	24.06	50.78	23.43	2.11	0.46
260	7	0.15	4.24	374.79	15.12	32.46	13.48	2.15	0.42

The hydrothermal carbonization treatment involves a decrease in pH value of the liquid phase at all conditions in the range of 3.69–4.49, mainly due to the production of organic acids (acetic, lactic, propionic, levulinic and formic acids) from the thermal decomposition of hemicellulosic sugars^16^. As observed by Hoekman et al.^56^ for the HTC liquor from HTC treatment of different woody and herbaceous biomass, the pH slightly decreases with increasing temperature. The same trend is obtained for the S:L ratio, due to the dilution effect of the water content, while the effect of residence time is not significant.

The analysis of the total organic carbon in the acqueous phase highlights that HTC treatment causes a significant increase from 9.71 g/L of the vinasse to the range of 12.05–22.93 g/L, with the highest value obtained at 260 °C, 4 h, and S:L ratio of 25. No strong effect of HTC temperature is observed with an initial decrease in TOC values up to about 220 °C, followed by a gradual increase due to the degradation of intermediates, such as organic acids compounds and sugars, to gas products. On the other hand the solid to liquid ratio has a strong impact on TOC yield, with a clear increase of TOC values with increasing S:L ratio. Also carbonization time plays a significant role in the TOC content of the spent liquor, in fact longer residence time gives slightly higher TOC results, demonstrating higher concentration of intermediates. The comparison of the obtained results for TOC in spent liquor with Literature data is difficult because, as reported by Mihajlovic et al.^57^, the trends of TOC values may differ depending on the type of biomass and operating conditions. COD values shows the same dependence of the TOC values on the HTC operating conditions. A linear relationship can be identified with COD values being much more than TOC, demonstrating the presence of inorganics in the spent liquor^58^. Moreover, the COD/TOC ratio shows high values, ranging from 1.91 to 2.72, indicating that the liquid phase at all HTC conditions contained a high concentration of oxidizable organics (sugars, organic acids)^59^. It is also interesting to note that the BOD/COD ratios are in the range 0.37–0.54, suggesting that the dissolved organics in the spent liquors are adequately biodegradable to be treated and valorized by biological methods^39^.

As regards the total nitrogen content in the liquid phase, a negligible effect of the residence time is observed, while the solid-to-liquid ratio and the temperature has a significant impact. In particular, it is interesting to analyze the effect of the HTC temperature; at the first temperature range (180–230 °C), the nitrogen yield in the spent liquor showed significant decrease, while during the second temperature range (230–260 °C), the total N shows slight increase, mostly due to the hydrothermal denitrification reaction and more inorganic nitrogen is produced^60^.

### Process optimization and validation

The desirability function approach^26^ is employed to determine the optimal conditions of the co-hydrothermal carbonization of winery and distillery by-products. The aim of the optimization process is to find the values of the HTC temperature, residence time and solid-to-liquid ratio in order to achieve maximal mass yield, energy densification yield, fuel ratio and phenols extraction yield and minimum energy consumption, simultaneously.

At this regard, data about the energy input at different operating conditions are shown in Table 2; the most significant contribution is the heating of the water, accounting in the range from 57 and 91% of the HTC total energy input, confirming that water is essential for the HTC treatment but heating it consumes energy. By applying the RSM optimizer tool of the Minitab software, every response is converted to dimensionless desirability value, *d*, and its value ranged between 0 and 1. Desirability is 1 if the response variable is at its goal, while is zero if the it is outside the acceptable range. Then, the goals are combined into an overall desirability function. Equal weights and importance are assigned for all the output responses due to high significance for all the responses that contribute to the HTC process.

Figure S6 (Supplementary Materials) shows the RSM optimizer generated results by the Minitab software. The low value of the maximum composite desirability (D = 0.50) demonstrates that it is difficult to optimize all the response variables simultaneously. The values of individual desirability indicates that the optimum parameters settings is more effective for maximizing PEY, followed by EDY, FR, EI and MY. The best optimized conditions are found to be 246.3 °C, 1.6 h, and a liquid-to-solid ratio of 0.066, while the predicted PEY, EDY, FR, and MY at these conditions are 21.88 mg GAE/g, 1.21, 0.60, and 52.64%, respectively.

Confirmation tests are also conducted at the optimal conditions by running three replicates in order to validate the accuracy of the predictive model. The results are PEY = 21.01 ± 0.34 mg GAE/g, EDY = 1.24 ± 0.05, FR = 0.61 ± 0.03, and MY = 50.77 ± 0.43%. The results indicates that the developed models are in agreement with experimental test results and can predict the responses within a 5% error. Furthermore, in order to evaluate the influence of vinasse as the moisture source for HTC process of winery and distillery by-products, experimental test at the optimal conditions is carried out by using deionized water as the moisture source. Due to the increasing water scarcity, it is essential to find alternative carbonization liquid sources. The experiments are conducted in triplicate and the mean values of the response variables are shown in Table 5.

**Table 5:** Physicochemical characterization of hydrochar and responses variables at the optimal HTC conditions, obtained using vinasse or deionized water as the reaction media.

	Vinasse	Deionized water
**Physicochemical characterization**		
C (wt%, db)	60.86	61.72
H (wt%, db)	7.09	6.76
N (wt%, db)	2.14	1.98
S (wt%, db)	0.22	0.14
O (wt%, db)	29.69	29.40
Volatile matter (wt%, db)	59.21	59.40
Ash (wt%, db)	3.58	3.33
Fixed carbon (wt%, db)	35.43	36.83
LHV (MJ/kg, db)	22.26	22.63
**Response variables**		
MY (%)	52.64	53.83
EDY	1.21	1.23
FR	0.60	0.62
PEY (mg GAE/g)	21.88	22.06

*LHV* lower heating value, *MY* hydrochar yield, *EDY* energy densification yield, *FR* fuel ratio, *PEY* phenols extraction yield.

It can be noted that hydrochar and spent liquor properties are substantially unaffected by carbonization in the presence of vinasse, according to the results obtained by Li et al.^21^, using landfill leachate and activated sludge as liquid source. The slight decrease in mass yield in the presence of vinasse, as compared to deionized water, could be attributed to the reactive constituents in the vinasse, such as organics, causing greater dissolution and hydrolysis of the biomass^20^. Therefore, the use of vinasse as liquid source does not have any negative impacts on the HTC process of winery and distillery by-products.

## Conclusions

The optimal conditions for the hydrothermal carbonization of winery and distillery by-products (vine pruning, EGM and vinasse) are found with regard to temperature, residence time and biomass-to-liquid ratio, by using RSM design, in order to concurrently produce a high-quality hydrochar, a liquor enriched in bioactives, such as phenols, and minimizing the energy consumption of the process. At optimized conditions, the temperature, holding time, and biomass-to-liquid ratio are 246.3 °C, 1.6 h, and 0.066, respectively, with 52.64% mass yield, 0.60 fuel ratio, 1.21 energy densification yield, and 21.88 mg GAE/g phenols extraction yield. Study results show that HTC temperature has the most influence in the physicochemical properties of hydrochar while the effects of S:L ratio and, in particular, residence time are less significant. In addition the use of vinasse, as moisture source, has no apparent negative impact on the HTC process efficiency both on the energy characteristics of the hydrochar and the phenols extraction efficiency. The results of this work demonstrate a circular and environmental-friendly approach for the waste management of the winemaking industry. The proposed value chain could have multiple advantages from the economic, environmental and social point of view. From the economic perspective, reduction costs of the management of winemaking and viticulture wastes could be achieved as well as their higher valorization in the energy and biochemical industries than the actual situation. On the environmental side, the opportunity to recover all wastes generated by the entire wine value chain could make winemaking industries more responsible for management aspects such as pollutant emissions, resource consumption and energy consumption. Finally, from the social point of view, the activation of this value chain could both improve the quality of life and rural development, ensuring jobs, and encourage the population and local authorities regarding environmental protection and recovery of viticulture by-products.

## Supplementary Information

Supplementary Information.

## Data Availability

All data generated or analysed during this study are included in this published article (and its Supplementary Information files).

## Abbreviations

BOD: Biochemical oxygen demand
COD: Chemical oxygen demand
EGM: Exhausted Grape Marc
HTC: Hydrothermal carbonization
HHV: Higher heating value
LHV: Lower heating value
RSM: Response surface methodology
TOC: Total organic carbon
BBD: Box–Behnken design
MY: Hydrochar yield
MH: Dry solid mass of hydrochar
MF: Dry solid mass of initial feedtostock
EDY: Energy densification yield
FR: Fuel ratio
VM: Volatile matter
FC: Fixed carbon
PEY: Phenols extraction yield
TPC: Total phenols content
ANOVA: Analysis of variance
EI: Energy input
H_water_: Enthalpy of water
c_p_: Specific heat capacity
A_s_: Surface area of the reactor
k: Thermal conductivity of the insulation material
s: Thickness of the insulation material
LOF: Lack of fit
GAE: Gallic acid equivalent
