# Supplementary material for: Valorization of winery and distillery by-products by hydrothermal carbonization
Source: Sci Rep. 2021 Dec 14;11:23973. doi: 10.1038/s41598-021-03501-7 (PMC8671441; doi:10.1038/s41598-021-03501-7)
Supplement: Supplementary file 1 — Supplementary Information. [file 41598_2021_3501_MOESM1_ESM.pdf]

Supplementary Materials

Valorization of winery and distillery by-products by hydrothermal carbonization

Marco Barbanera[1,*], Alessandro Cardarelli[1], Eleonora Carota[2], Marco Castellini[1], Tommaso Giannoni[3], Stefano Ubertini[1]

[1]*Department of Economics Engineering Society and Business Organization (DEIM), University of Tuscia, Largo dell'Università s.n.c., Loc. Riello, 01100 Viterbo, Italy*

[2]*Department for Innovation in Biological, Agro-food and Forest Systems (DIBAF), University of Tuscia, Viterbo, 01100, Italy*

[3]*CIRIAF—Biomass Research Centre, University of Perugia, Via G. Duranti 63, 06125 Perugia, Italy*

* Corresponding author: Marco Barbanera

Source	F-value	p-value
MY [%]		
Model	47.61	<0.0001
A	360.26	<0.0001
B	12.85	0.016
C	46.99	0.001
A^2	0.28	0.622
B^2	0.74	0.430
C^2	1.13	0.337
AB	2.07	0.209
AC	1.84	0.233
BC	2.63	0.166
Lack of fit	2.62	0.289
R^2 = 0.9885, R^2adj = 0.9677, R^2pred = 0.8477		
EDY		
Model	134.72	<0.0001
A	1035.54	<0.0001
B	143.81	<0.0001
C	10.42	0.023
A^2	11.65	0.019
B^2	0.28	0.620
C^2	4.25	0.094
AB	1.64	0.256
AC	0.02	0.991
BC	3.57	0.118
Lack of fit	16.72	0.057
R^2 = 0.9959, R^2adj = 0.9885, R^2pred = 0.9365		
FR		
Model	27.81	0.001
A	213.66	<0.0001
B	30.55	0.003
C	0.01	0.998
A^2	0.69	0.443
B^2	1.29	0.308
C^2	0.24	0.643
AB	0.74	0.430
AC	0.07	0.808
BC	2.87	0.151
Lack of fit	8.86	0.103
R^2 = 0.9804, R^2adj = 0.9452, R^2pred = 0.8809		
PEY [mg GAE/g]		
Model	33.34	0.001
A	66.44	<0.0001
B	0.07	0.798
C	175.16	<0.0001
A^2	0.02	0.892
B^2	0.17	0.701
C^2	57.16	0.001
AB	0.03	0.867
AC	0.64	0.459
BC	0.36	0.576
Lack of fit	2.91	0.266
R^2 = 0.9836, R^2adj = 0.9541, R^2pred = 0.8898		

Table.S1. ANOVA of response surface quadratic models (MY: Hydrochar Yield; EDY: Energy Densification Yield; FR: Fuel Ratio; PEY: Phenols Extraction Yield)

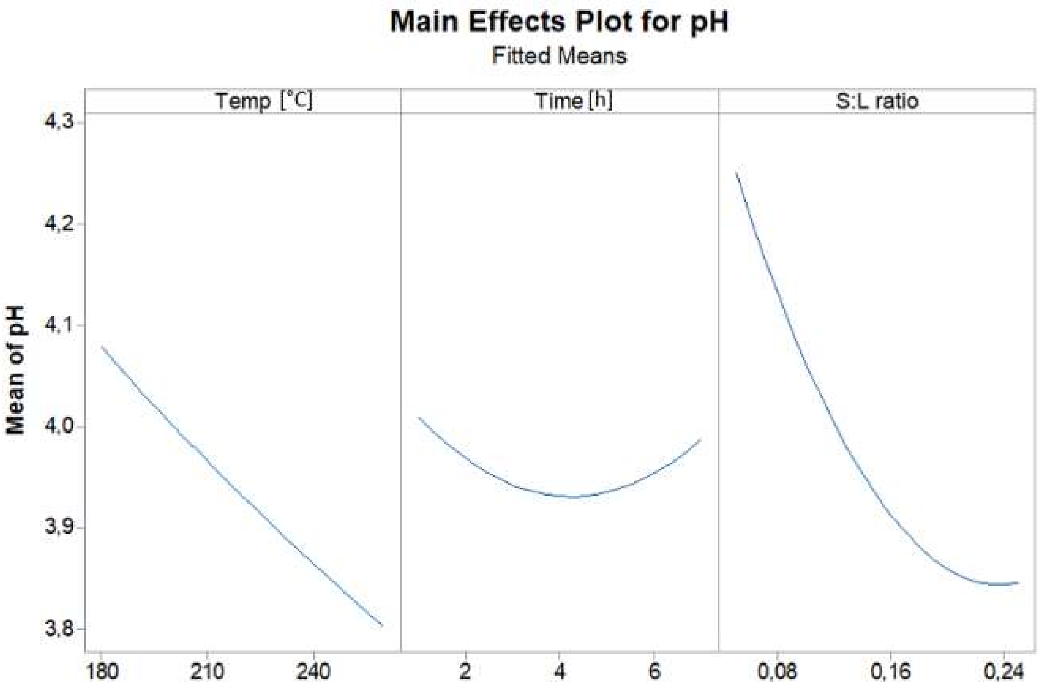

Figure. S1. Main effects plot for pH.

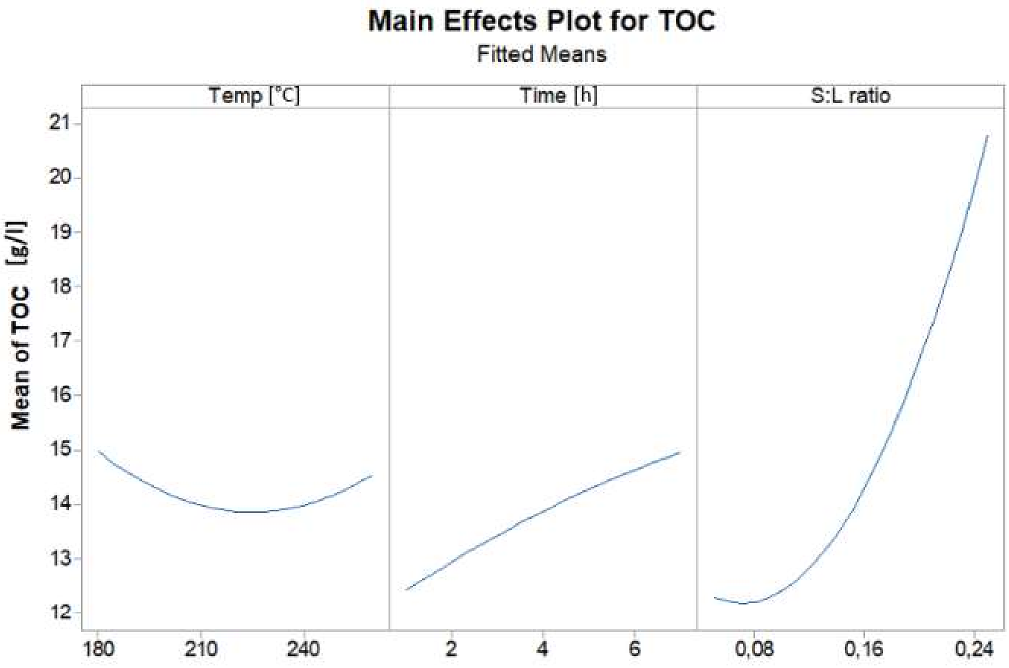

Figure. S2. Main effects plot for TOC.

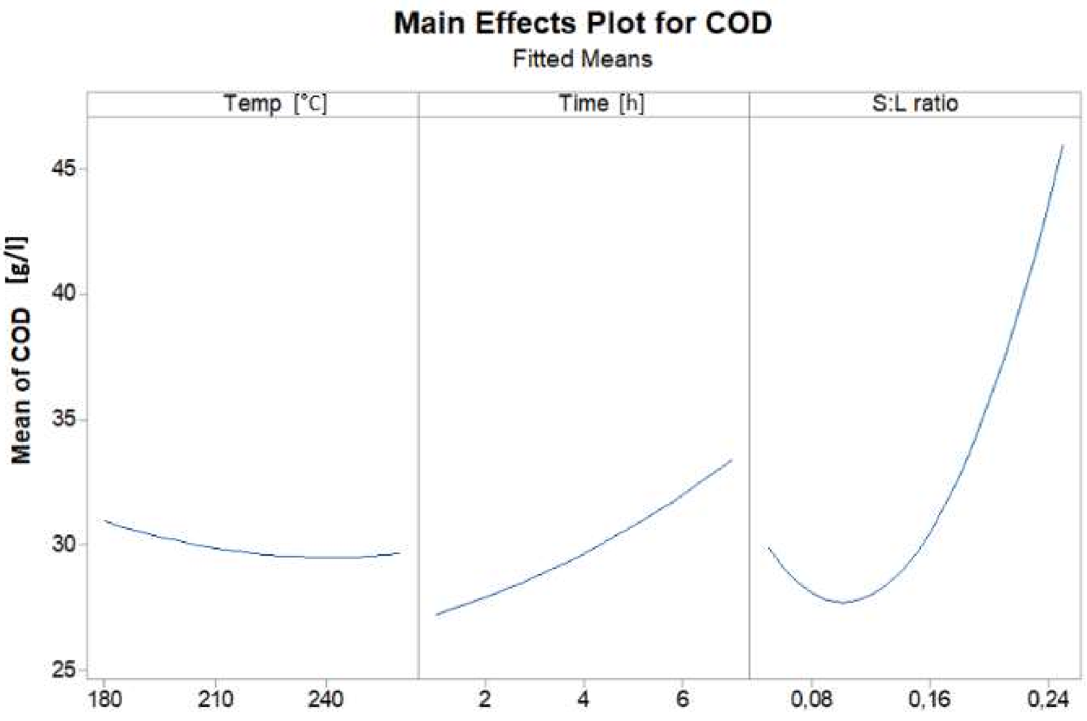

Figure. S3. Main effects plot for COD**.**

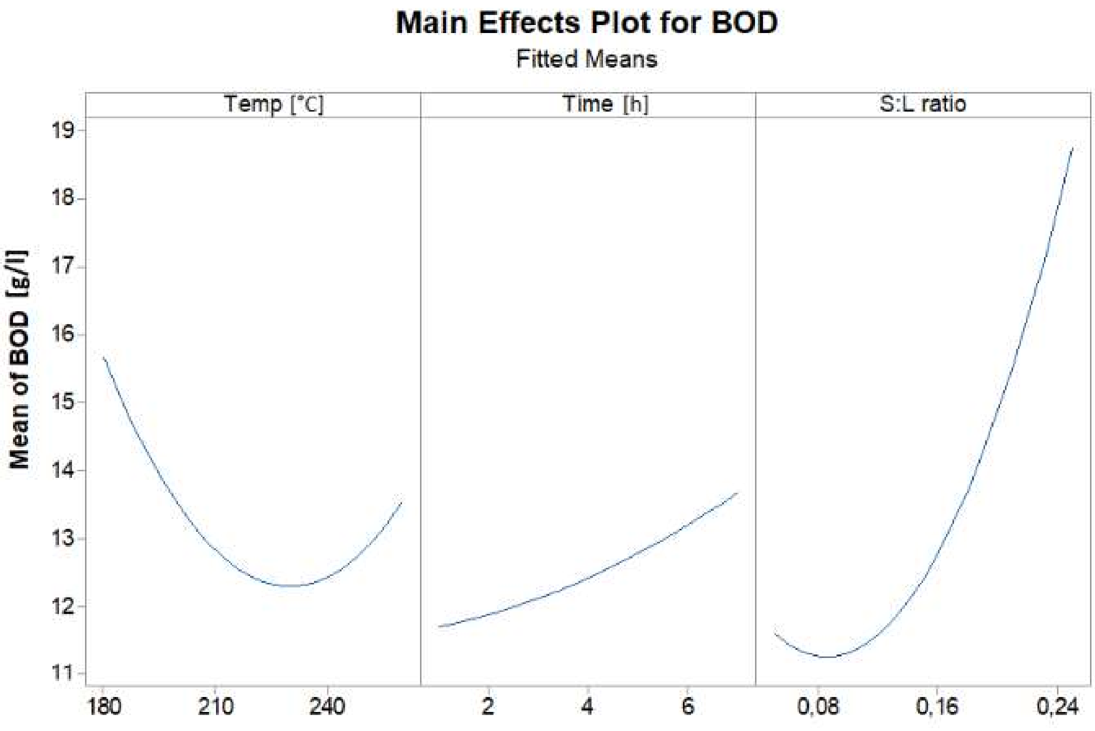

Figure. S4. Main effects plot for BOD**.**

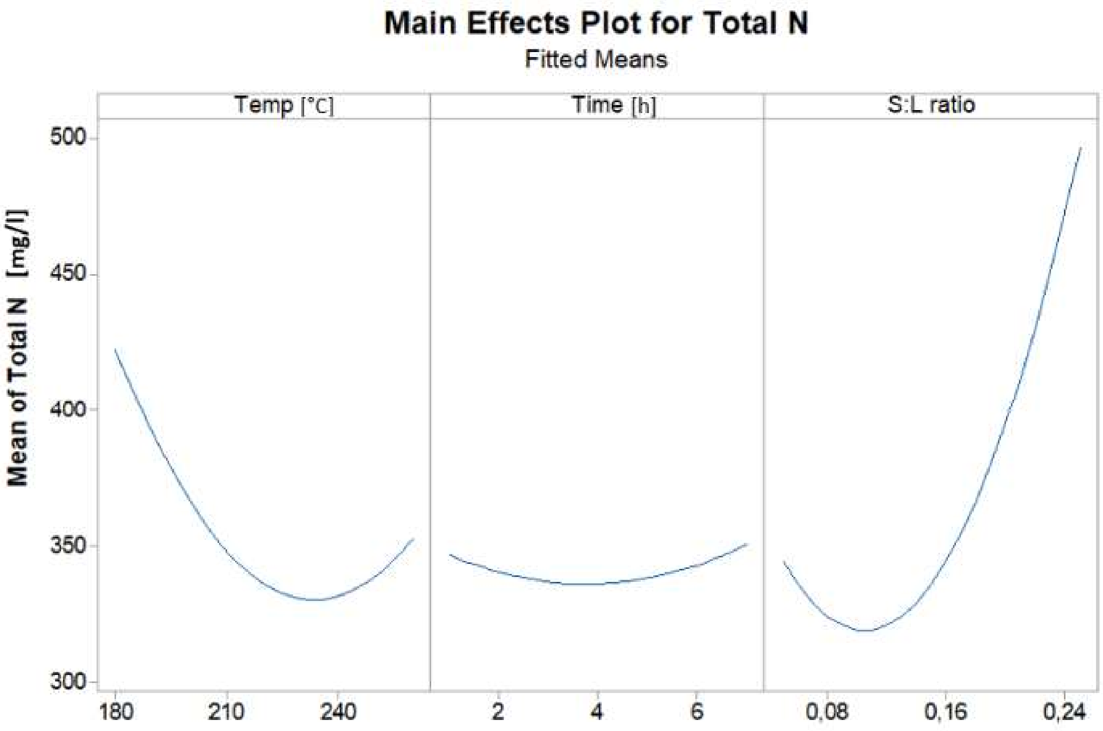

Figure. S5. Main effects plot for total nitrogen**.**

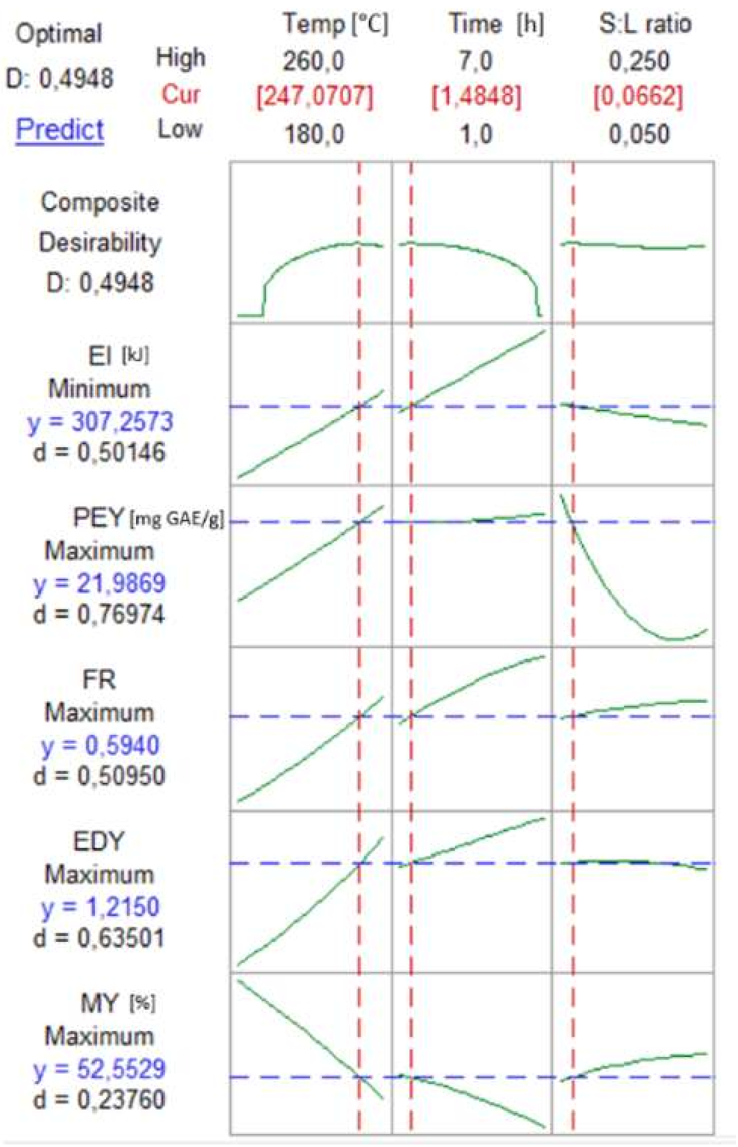

Figure. S6. RSM optimizer plot (MY: Hydrochar Yield; EDY: Energy Densification Yield; FR: Fuel Ratio; PEY: Phenols Extraction Yield; EI: Energy Input).
